# Geospatial analysis of childhood morbidity in Ghana

**DOI:** 10.1371/journal.pone.0221324

**Published:** 2019-08-30

**Authors:** Anthony Mwinilanaa Tampah-Naah, Adams Osman, Akwasi Kumi-Kyereme

**Affiliations:** 1 Department of Environment and Resource Studies, Faculty of Integrated Development Studies, Wa Campus, University for Development Studies, Tamale, Ghana; 2 Department of Geography and Regional Planning, College of Humanities and Legal Studies, University of Cape Coast, Cape Coast, Ghana; 3 Department of Population and Health, College of Humanities and Legal Studies, University of Cape Coast, Cape Coast, Ghana; The University of Hong Kong, CHINA

## Abstract

**Introduction:**

Childhood morbidities are common in Ghana. The present study sought to geospatially analyze morbidities among children (0–23 months of age) using five different survey datasets (1993–2014) from the Ghana Demographic and Health Survey.

**Methods:**

Logistic regression was used to examine childhood morbidity within a place of residence. Then three spatial statistical tools were applied to analyze morbidities among children (0–23 months of age). These tools were: spatial autocorrelation (Global Moran’s *I*)—used to examine clustering or dispersion patterns; cluster and outlier analysis (Anselin’s local Moran’s *I*)—to ascertain geographic composition of childhood morbidity clusters and outliers; and hot spot analysis (Getis-Ord G)—to identify clusters of high values (hot spots) and low values (cold spots).

**Results:**

Children in rural areas were much burdened with the occurrence of childhood morbidity. The study revealed positive spatial autocorrelation for childhood morbidity in Ghana. Childhood morbidity (diarrhoea, ARI, anaemia, and fever) clusters were identified within districts in the country. Children in rural areas were more likely to be morbid with diarrhoea, anaemia, and fever compared to those in urban areas. Hot spot districts for diarrhoea, anaemia and fever were mainly situated in semi-arid areas and those with ARI were located both at the semi-arid areas and coastal portions of Ghana.

**Conclusion:**

Rural children are much exposed to have higher burden of a childhood morbidity compared to their urban counterparts. Most semi-arid districts in Ghana are burdened with diarrhoea, ARI, anaemia, and fever. To minimize the occurrence of childhood morbidity in Ghana, designing of more context-based interventions to target hot spots districts of these morbidities are required in order to use scarce resources judiciously.

## Introduction

The health of children requires much attention since occurrence of morbidity during this tender age could have lifelong effects on their growth and development [1, 2]. To improve the health of children, it is necessary for health planners to effectively comprehend the spatial homogeneity and heterogeneity of top rated childhood morbidities. Children in developing countries, places with low socio-economic indicators or poor environmental conditions, commonly suffer from diarrhoea [3], respiratory infections [4], anaemia [5] and fever [6]. Diarrhoea is one of the major causes of mortality in children under five years old, and it accounts for about 760,000 mortality cases every year [7]. Approximately, four to five million children each year in developing countries die due to acute respiratory infections (ARI) [8]. Also, prevalence of anaemia was estimated to be 65 percent in Africa (in 2008) [9], and fever is a common symptom of most childhood morbidities [10].

In Ghana, diarrhoea, ARI, anaemia, and fever are also the leading causes of morbidity and mortality among children [11]. The prevalence of these childhood morbidities among under 5 years showed that 12 percent had all forms of diarrhoea, four percent had ARI symptoms, 66 percent (of children 6–59 months) were anaemic and 14 percent (of children under five) had fever in 2014 [12].

Elsewhere, empirical evidences indicate that studies have been conducted to establish hot spots of morbidities among children [13, 14]. Similarly in Ghana, a number of authors have examined spatio-temporal patterns of morbidities using facility-based data [15, 16, 17, 18]. All these studies commonly examined a single morbidity such as diarrhoea among children. In addition, a number of studies either selected towns or a small number of districts to spatially assess morbidities. The usage of more layers (thus all districts) in our study represents an increase in scale compared with the limited studies on spatial analysis of childhood morbidities in the country. We examined spatial clustering and hot spots of four most reported morbidities among infants and young children in Ghana (aged 0–23 months of age). Findings of the study may inform related stakeholders (such as Ghana Health Services and health related non-governmental organisations) on how to programme and allocate their resources effectively. The study, therefore, sought to geospatially analyse concurrently four the morbidities among children using five datasets (2014, 2008, 2003, 1998, 1993) from the Ghana Demographic and Health Surveys (GDHS).

## Methods

### Study setting

**Fig 1:**
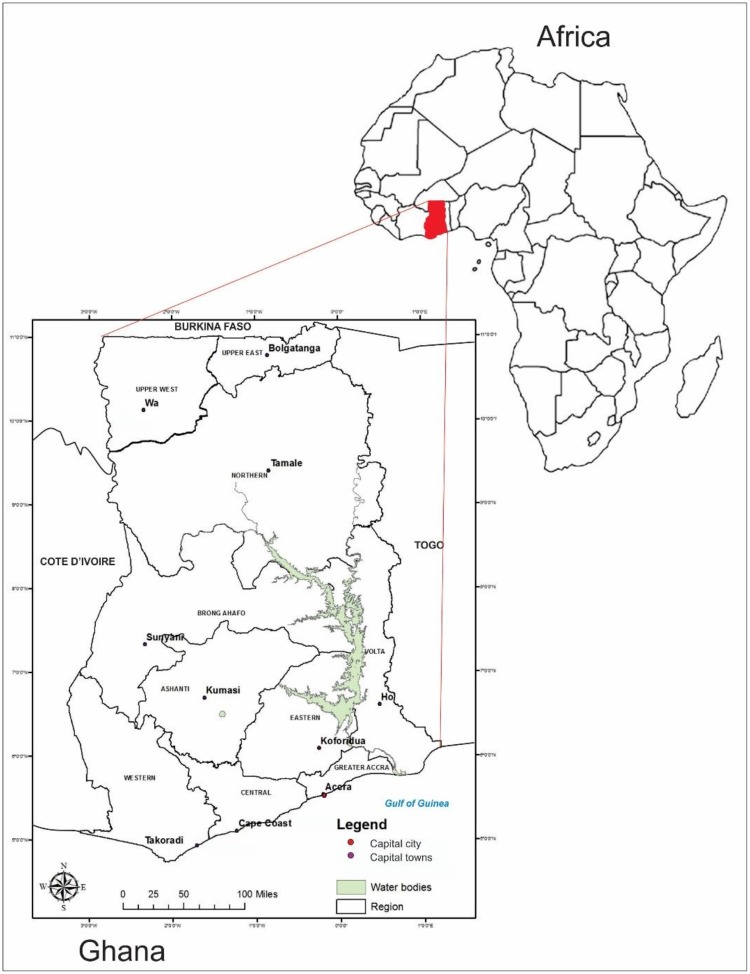
Map of Africa projected to show map of Ghana. Ghana is located in the Western portion of Africa. The country is boarded to the: north by Burkina-Faso; west by Cote D’Ivoire; east by Togo; and south by Gulf of Guinea.

Ghana lies between latitudes 4°45’N and 11°N, and longitudes 1°15’E and 3°15’W. The country is located in West Africa with a total land area of 238,537 square kilometres; and bordered by three countries: to the east by Togo; Cote d’Ivoire to the west; Burkina Faso to the north and south by the Gulf of Guinea (Fig 1). The population of the country as recorded in the 2010 Population and Housing Census was 24,658,823 million people with an average annual growth of about 2.5 percent. Ghana is divided into ten main administrative regions with 216 sub-units (metropolis/municipal and districts) [12].

### Data source

The study used five different GDHS datasets (2014, 2008, 2003, 1998, 1993). These data were acquired from Demographic and Health Survey (DHS) programme website (https://dhsprogram.com/). The GDHS is a national-level population and health survey conducted in Ghana as part of the Global Demographic and Health Survey programme; and the surveys are conducted in the country in partnership with Ghana Statistical Service and Ghana Health Service. STATA data files (with individual rows containing health related details on women [15–49 years] including their children), and shapefiles for the location of clusters visited (during the surveys) were specifically used for this study. The data is available at https://www.dhsprogram.com/data/available-datasets.cfm. For each of the surveys conducted in the country, approval was sought from Ghana Health Service Research Ethical Committee.

### Sampling technique

The GDHS used a two-stage stratified nationally representative sample of households [12]. All the surveys usually used the previous sample points (clusters) comprising of enumeration areas of national population censuses as the sampling frame to produce separate estimates for key indicators for the ten administrative regions in the country. Sample points (clusters) were situated within districts, and the primary sampling units consisted both urban and rural clusters. The secondary sampling units included households. Households were listed in all enumerated areas and they were randomly selected from the list. A number of households were randomly selected from each cluster (found within districts) and different sample sizes were obtained at the end of each sample design for each survey period. More details on the sampling techniques used for each survey can be obtained from the various survey report documents at https://dhsprogram.com/Publications/Publications-by-Country.cfm.

### Data collection

In collecting the household data, enumerators also mapped the clusters in which they interviewed respondents. The clusters were mapped, rather than the house of the respondent, to protect actual identity and location of respondents. However, the mapped spatial clusters have primary fields which enable easy data merging with household records. Spatial data of the boundaries of 216 administrative sub-units were solicited from the Ghana Survey Department to enable the analysis to be made at a district level. We analysed at the district level because this level is more informative to policy makers rather than analysing at the cluster level.

### Data preparation

The study used GDHS mapped clusters, and 216 district administrative layers. The 216 administrative layers had their geographic coordinates as the Ghana Meter Grid (projected coordinate system) while clusters mapped from GDHS was in the World Geodetic System 1984 (geographic coordinate system). The GDHS spatial data was re-projected into the projected coordinate system of Ghana Meter Grid. The two spatial datasets were then later converted from shapefile to ERSI geodatabase format; this was done using ArcGIS 10.3. Then a spatial join was undertaken to transfer cluster names to the sub-administrative polygon layer. Some of the extracted sub-administrative units had more than one cluster. Such clusters were aggregated and their means were used to represent the respective district they fell within.

For the prevalence of childhood morbidity (diarrhoea, ARI, anaemia, and fever), data was extracted from the individual datasets from the various surveys. The extraction was restricted to children less than two years who were living with their mothers during the survey periods. This category of children is deemed to be more susceptible to episodes of morbidities compared to children more than two years. Mothers were asked whether any of their children under five years of age had diarrhoea during the two weeks preceding the survey. ARI was estimated by asking mothers whether their children under age five had been ill with a cough accompanied by short rapid breathing in the two weeks preceding the survey. For anaemia, children who stayed in the household on the night before the survey were tested for anaemia based on their haemoglobin levels. With fever, mothers were asked whether a child has been ill with fever at any time in the last two weeks preceding the survey. All the survey years contained the required variables of interest except surveys of 1993, and 1988 that did not have anaemia data. Extracted data on child morbidity were spatially joined with the sub-administrative unit polygon to generate geo-relational data to enable spatial analysis. Stata version 12 was used to process and extract the childhood morbidity.

### Statistical analyses

Logistic regression was used to examine association between childhood morbidity and place of residence by adjusting for the effect of other covariates (place of residence, education, working status, and drinking water. These variables can potentially influence health outcomes in children. This was done to further explain the occurrences of childhood morbidity in the districts found to have significant geographic clusters. The place of residence was considered as the main independent variable since the GDHS data were mainly collected within clusters found within districts that could be either be described as an urban area or a rural area. The other covariates were selected based on their possible effects across all the childhood morbidities. For each morbidity, two models (unadjusted and adjusted) were generated. Results were presented as odds ratio and 95 percent confidence intervals.

For the geospatial analyses, three spatial statistical tools were applied to analyze the data. These tools are: spatial autocorrelation (Global Moran’s *I*); cluster and outlier analysis (Anselin’s local Moran’s *I*); and hot spot analysis (Getis-Ord G). Spatial autocorrelation (spatial association) measures clustering or dispersion based on feature geographical locations and attribute values of a single variable. It is used to calculate correlation among neighbouring observations and to ascertain patterns and levels of spatial clustering in neighbouring districts [19, 20]. It computes a single summary value, a z-score (of -1 to +1), describing the degree of spatial concentration or dispersion for a measured variable [21].

The spatial autocorrelation was, therefore, used to examine whether childhood morbidity (diarrhoea, ARI, anaemia, and fever) had a clustering or dispersion pattern in the country using districts as features. The study hypothesized that the prevalence of study phenomena (childhood morbidity) are randomly distributed (no spatial dependency) across various districts in the country. The null hypothesis is rejected if a calculated *p*-value is very small (95% confidence interval); which implies an unlikely situation that observed spatial pattern is as a result of random processes. Hence, a district with a high *z*-score and a small *p*-value indicates a spatial clustering of high values.

To locate where childhood morbidity was clustered, the ArcGIS clusters and outlier analysis tool was applied. Cluster and outlier tool measures spatial autocorrelation based on both area locations and area values simultaneously. Given a set of areas and an associated attribute, it evaluates whether the pattern expressed is clustered, dispersed, or randomized [22].

The cluster and outlier analysis (Anselin Local Moran’s *I*) was used to ascertain geographic composition of childhood morbidity clusters and outliers. This statistic calculates a *z*-score, a *p*-value, and a code representing each cluster type into four statistical significant outcomes. High positive z-score for a district means that its surrounding districts have similar values, High-High (districts showing high levels of morbidity surrounded by districts with similar high levels) for a statistically significant (0.05 level) cluster of high values and Low-Low (districts showing low levels of morbidity surrounded by districts with similar low levels) for a statistically significant (0.05 level) cluster of low values. Low negative z-score for a district means a statistically significant (0.05 level) spatial outlier, High-Low for a district a high value surrounded by low values Low-High for low value surrounded by high values [22]. The Moran’s *I* identifies clusters of features with values similar in magnitude. Moran’s *I* values range from -1 (disperse) to +1 (clustered). A calculated Moran’s *I* value of 0 indicates complete spatial randomness. This study used *p* < 0.05 to determine statistical significance of the computed index values.

Further, hot spot analysis measures each area in a dataset within the context of neighbouring areas in the same dataset. It uses vectors to identify areas of statistically significant hot spots and cold spots in data. Also, hot spot analysis assumes that there is the presence of clustering within the data [23]. Hot spot analysis is used to identify statistically significant spatial clusters of high values (hot spots) and low values (cold spots). This analysis was used to define districts with high prevalence versus districts of low prevalence of childhood morbidity. The description of a district as being a hot spot of a childhood morbidity was expressed using 99%, 95% and 90% confidence levels (CL). The usage of these informs the reader about the prominence of hot spots. The CL indicates the significance of occurrence of a morbidity in a district. Only districts identified as hotspots at the 99% confidence level (high-lighted table in the results section) and then while all hot spots were represented on the maps.

The three analyses all adopted Inverse Distance Method (IDM) with the notion that near incidents are more related than those afar. However, the False Discovery Rate Correction (FDRC) was not applied. This was to allow the analysis to be conducted based on individual district first in isolation.

## Results

Table 1 shows the association between childhood morbidity and selected covariates after controlling some other socio-demographic factors. Children in rural areas (OR = 1.32, 95% CI = 1.25–1.52) were more likely to have had experienced diarrhoea compared to children in urban areas. After adjusting for other factors, the odds of children in rural areas to experience episodes of diarrhoea was still positive significant (OR = 1.19, 95% CI = 1.39). In addition, children with mothers who attained secondary or higher education (OR = 0.75, 95% CI = 0.64–0.88) were less likely to report occurrence of diarrhoea compared to those whose mothers had no formal education. Likewise, mothers who had access to unimproved sources of drinking water (OR = 0.82, 95% CI = 0.71–0.96) reported less occurrence of childhood diarrhoea compared to those who has access to improved sources of drinking water. For ARI, no significant associations were found with the covariates.

**Table 1:** Association between childhood morbidity and covariates.

Covariates	Diarrhoea		ARI		Anaemia		Fever	
	M 1	M 2	M 1	M 2	M 1	M 2	M 1	M 2
**Place of Residence**								
Urban	Ref.	Ref.	Ref.	Ref.	Ref.	Ref.	Ref.	Ref.
Rural	1.32*(0.00) [1.15–1.52]	1.19*(0.03) [1.02–1.39]	0.99(0.89) [0.87–1.13]	0.94(0.42) [0.82–1.09]	1.41(0.00) [1.23–1.61]	1.37*(0.00) [1.17–1.59]	1.29*(0.00) [1.22–1.48]	1.16*(0.05) [0.99–1.36]
**Education**								
No education		Ref.		Ref.		Ref.		Ref.
Primary		0.95(0.59) [0.797–1.14]		1.11(0.26) [0.93–1.32]		1.03(0.76) [0.85–1.24]		0.92(0.35) [0.76–1.09]
Secondary/Higher		0.75*(0.00) [0.64–0.88]		1.01(0.94) [0.86–1.17]		0.88(0.12) [0.74–1.04]		0.83(0.02) [0.71–0.97]
**Working status**								
Not working		Ref.		Ref.		Ref.		Ref.
Working		1.19(0.06) [0.71–0.96]		1.13(0.15) [0.96–1.33]		1.04(0.68) [0.86–1.25]		1.47*(0.00) [1.22–1.77]
**Drinking water**								
Improved		Ref.		Ref.		Ref.		Ref.
Unimproved		0.82*(0.01) [0.71–0.96]		1.02(0.83) [0.88–1.17]		0.96(0.57) [0.82–1.12]		0.95(0.47) [0.81–1.09]

M-model; ARI-acute respiratory infection; Ref.-reference group;

*-significant at p<0.05

p-values are in round brackets, and 95% confidence interval ranges are in square brackets

Children in rural areas (OR = 1.37, 95% CI = 1.17–1.59) were more likely to be anaemic compared to their counterparts in urban areas (Table 1). This significance was still there after holding other factors constant. More feverish children were likely to be found in rural areas (OR = 1.29, 95% CI = 1.22–1.48) compared to urban areas. After adjusting for other covariates, children in rural areas were still more likely to experience fever compared to those in urban areas. Also, children whose mothers were working (OR = 1.47, 95% CI = 1.22–1.77) at the time of survey were likely to have more episodes of fever compared to those whose mothers were not working.

### Spatial autocorrelation of childhood morbidity

The study revealed positive spatial autocorrelation for childhood morbidity in the country (Table 2). In 2014, there were clustering of similar values for diarrhoea (Moran’s *I* = 0.096; *p* = 0.001), ARI (Moran’s *I* = 0.073; *p* = 0.004) and anaemia (Moran’s *I* = 0.056; *p* = 0.024), suggesting that there is less than 1 percent possibility that these clustered patterns could be attributed to random chance. In other words, the output was significant indicating clustering of acute respiratory infections at the district level. Also meaning, there were areas of hotspots in data. In 2008 and 2003, for diarrhoea, given the Moran’s *I* of 0.078 (*p* = 0.007) and 0.063 (*p* = 0.019) respectively, the patterns appeared to be significantly significant thus cases of diarrhoea were not randomly distributed among the districts. The clustered patterns of diarrhoea, ARI, and fever were statistically significant in 1998 and 1993; thus suggesting that the clustered patterns were not the result of random chance.

**Table 2:** Global Moran’s *I* analysis of childhood morbidity (1993–2014).

Morbidity	Year	Moran’s Index	Expected Index	Variance	*z*-score	*p*-value
Diarrhoea	2014	0.096479	-0.004651	0.000728	3.748256	0.000178*
ARI		0.072805	-0.004651	0.000726	2.874740	0.004044*
Anaemia		0.056534	-0.004651	0.000737	2.253649	0.024218*
Fever		0.028028	-0.004651	0.000724	1.214795	0.224444
Diarrhoea	2008	0.078913	-0.006024	0.001004	2.681021	0.007340*
ARI		0.027528	-0.006024	0.000973	1.075815	0.282010
Anaemia		0.014738	-0.006024	0.665518	0.665518	0.505719
Fever		0.017944	-0.000982	0.000982	0.764720	0.444438
Diarrhoea	2003	0.063113	-0.005747	0.000859	2.349601	0.018794*
ARI		0.041527	-0.005747	0.000877	1.596134	0.110459
Anaemia		0.041527	-0.005747	0.000877	1.596124	0.110459
Fever		0.026199	-0.005747	0.000877	1.078826	0.280665
Diarrhoea	1998	0.083797	-0.005650	0.000900	2.981013	0.002873*
ARI		0.067498	-0.005650	0.000865	2.486490	0.012901*
Fever		0.138308	-0.005650	0.000883	4.845580	0.000001*
Diarrhoea	1993	0.071159	-0.005780	0.000961	2.481752	0.013074*
ARI		0.052478	-0.005780	0.000830	2.022656	0.043109*
Fever		0.130203	-0.005780	0.000974	4.357366	0.000013*

ARI-Acute respiratory infection;

* statistically significant (p < 0.05)

Because of the low values for Moran’s *I* indicating low positive correlation necessitated further analysis to understand at what spatial scale the data cluster. An incremental analysis was performed.

Table 3 indicates that the spatial scale at which each childhood morbidity indicated positive spatial autocorrelation varied. Diarrhoea did not have any peak distance for positive correlation for 2014, 2008 but was significant in 1993 (with a peak distance of 17135.07meters. The remaining morbidity had peak positive spatial correlation within minimum distance of 101951.24 meters and maximum of 185659.79 meters.

**Table 3:** Incremental spatial autocorrelation Global Moran’s *I*’s analysis of childhood morbidity (1993–2014).

Morbidity	Year	Peak distance (m)	Moran’s Index	Expected Index	Variance	*z*-score	*p*-value
Diarrhoea	2014	-	-	-	-	-	-
ARI		117331.86	0.059704	-0.005291	0.000374	3.360480	0.000778 *
Anaemia		178854.34	0.039277	-0.005291	0.000161	3.514823	0.000440 *
Fever		101951.24	0.066769	-0.005291	0.000459	3.361907	0.000774*
Diarrhoea	2008	-	-	-	-	-	-
ARI		-	-	-	-	-	-
Anaemia		148214.05	0.080945	-0.006024	0.000293	5.083468	0.000000*
Fever		162562.52	0.102688	-0.005952	0.000239	7.034425	0.000000*
Diarrhoea	2003	139956.83	0.144554	-0.005747	0.000308	8.570825	0.000000*
ARI		139956.83	0.101640	-0.005747	0.000315	6.054973	0.000000*
Anaemia		139956.83	0.101640	-0.005747	0.000315	6.054973	0.000000*
Fever		139760.77	0.046944	-0.005682	0.000312	2.980574	0.002877*
Diarrhoea	1998	118649.23	0.107844	-0.005650	0.000418	5.552924	0.000000*
ARI		147790.71	0.072297	-0.005650	0.000263	4.804862	0.000002*
Fever		-	-	-	-	-	-
Diarrhoea	1993	171351.07	0.141159	-0.005780	0.000204	10.288151	0.000000*
ARI		171351.07	0.067507	-0.005780	0.000174	5.553500	0.000000*
Fever		185659.79	0.124020	-0.005780	0.000170	9.967209	0.000000*

-No peak distance results

* statistically significant (p < 0.05)

### Spatial clusters of childhood morbidity

Childhood morbidity (diarrhoea, ARI, anaemia, and fever) clusters were identified. The clustered districts were classified as: high-high cluster (light brown coloured); high-low outlier (red coloured); low-high outlier (blue coloured); and low-low cluster (light blue coloured). Clustering of childhood morbidity was found largely in the middle and northern portions of the country.

**Fig 2:**
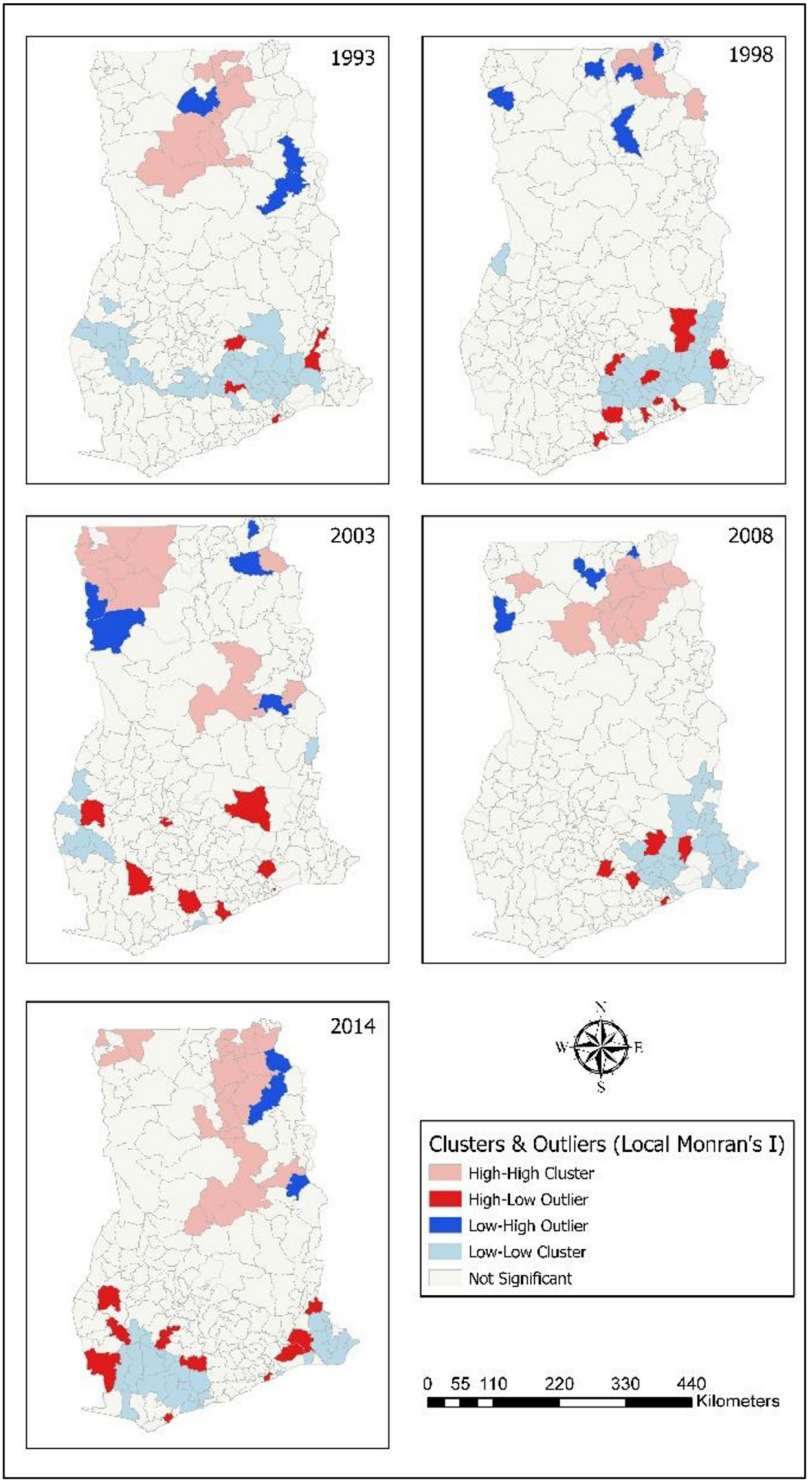
Spatial cluster analysis (Local Moran I) of childhood diarrhoea. 1993–Districts with high clusters of diarrhoea found were situated in Upper East, and Northern. 1998–High clustered districts were in Upper East, and Northern. 2003–High clustered districts were found in Upper West, and Northern. 2008–High clustered districts in Upper West, Upper East, and Northern. 2014–Districts with high clusters of diarrhoea located in Upper West, Upper East, Northern, and Brong Ahafo.

Most of the high clustered cases of diarrhoea where located in the Upper West, Upper East, and Northern. Except for the year 2003, most of the clustering occurred in Upper East, and Northern. In 2014, 19 districts were found to have highly clustered levels of diarrhoea cases. Fifteen of the highly clustered districts were found in Upper East (6) and Northern (9). A few of the highly clustered districts were found in Upper West (3), and Brong Ahafo (1) (Fig 2). Nine districts were found to have clustered occurrence of diarrhoea with Northern having more of them in 2008. Six districts had high clustered cases of diarrhoea in Upper East and Northern in 1998 and ten districts exhibited high clustered episodes of diarrhoea in 1993.

**Fig 3:**
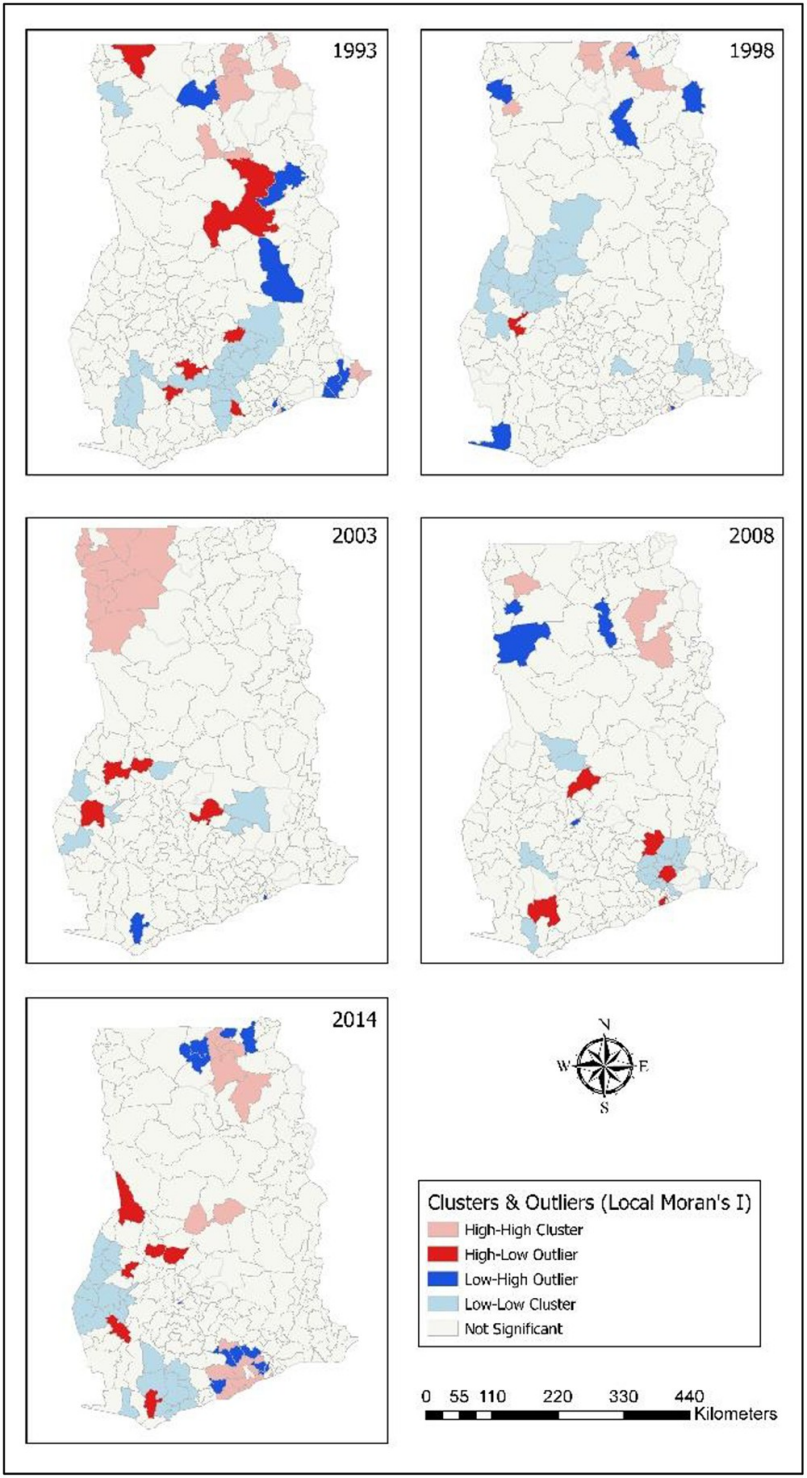
Spatial cluster analysis (Local Moran I) of childhood ARI. 1993–High clustered districts with ARI found in Upper East, Northern, and Volta. 1998–High clustered districts were in Upper East, and Northern. 2003–high clustered districts located in Upper West, and Northern. 2008–High clusters situated in Upper West, and Northern. 2014–Districts in Upper East, Northern, Brong Ahafo, Eastern, Accra, and Central showed high clusters of ARI.

Clustering of ARI happened more in the northern portion (Upper West, Upper East, Northern) and some occurred in the middle and southern portions (Central, Accra, Eastern) of the country. Fig 3 displays districts identified with high clustered cases of ARI. In 2014, five districts in Upper East, three in Eastern, two in Greater Accra and three in Central were identified to have high clustered occurrence of ARI. Also, high clusters of ARI were found in three districts in 2008; 11 districts in 2003; six districts in 1998; and 10 districts in 1993.

**Fig 4:**
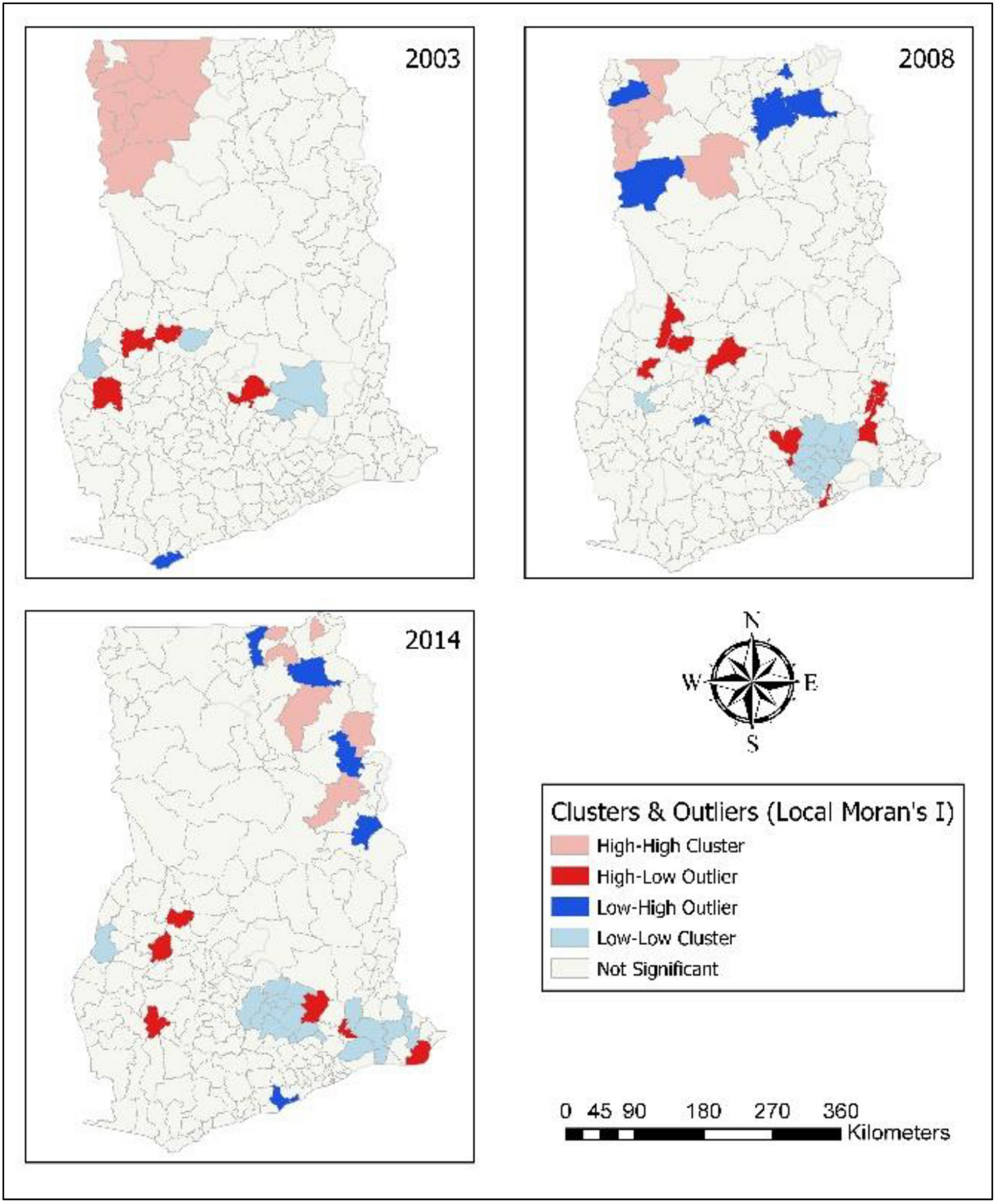
Spatial cluster analysis (Local Moran I) of childhood anaemia. 1993–High clustered districts in Upper East, Northern, Eastern, and Volta. 1998–High clustered districts in Upper West, Upper East, Northern, and Accra. 2003–All high clustered districts in Upper West. 2008–High clustered districts in Upper East, Northern, and Brong Ahafo. 2014–High clustered districts in Upper West, Upper East, Northern, and Brong Ahafo.

Most clustered cases of anaemia occurred in Upper West in the year 2003. For the following years, few clustered cases were still observed in Upper West in 2008 while some patches of clusters occurred in Northern, and Upper East in 2014. Also, six districts each were identified to have high clusters of anaemia in 2014 and 2008. In 2003, ten districts showed high clustered occurrence of anaemia in Upper West (Fig 4). Almost the districts Upper West showed high levels of clustering in 2003. Some clustering was observed in 2008 and 2014 for Upper West, Upper East, and Northern.

**Fig 5:**
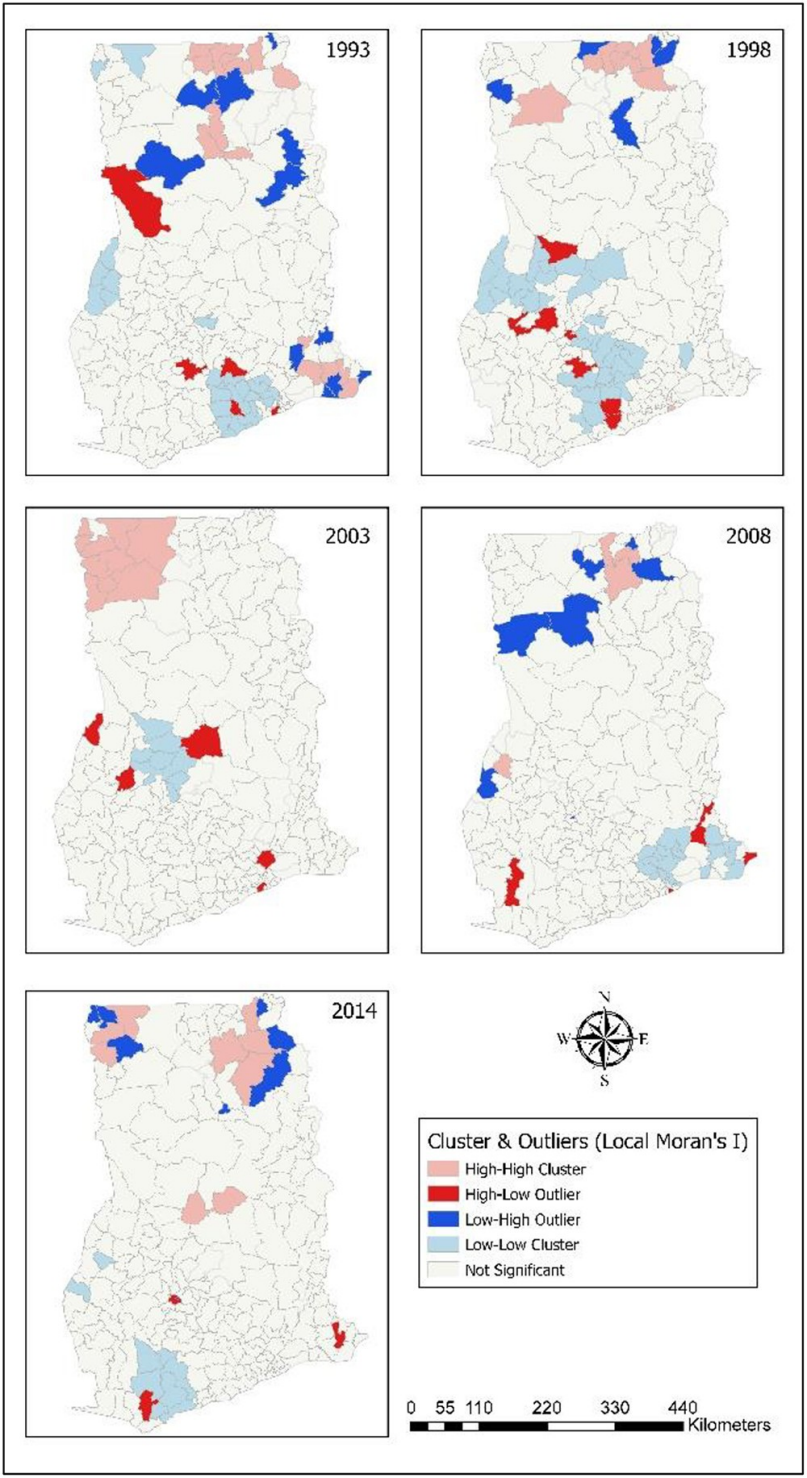
Spatial cluster analysis (Local Moran I) of childhood fever. 1993–Upper East, Northern, Eastern, and Volta had districts with high clusters of Fever. 1998–High clustered districts were in Upper East, Northern, and Accra. 2003–All clustered districts in Upper West. 2008—Upper East, Northern, and Brong Ahafo had high clustered districts. 2014–Clustered districts found in Upper West, Upper East, Northern, and Brong Ahafo.

Across the years, more clusters of fever were in Upper West for the year 2003. For the other years, high clusters of fever were found in Upper East, Northern, and Brong Ahafo. In relation to fever, eight districts displayed high clusters in 2014 (Fig 5). In the years 2008 and 2003, 10 districts demonstrated high clustering of children who experienced fever. Eleven districts displayed highly clustered levels of fever were found in 1998 and 17 districts equally were linked with clustered patterns in 1993. Most districts identified with fever were found within the northern portions of the country.

### Hot spots of childhood morbidity

**Fig 6:**
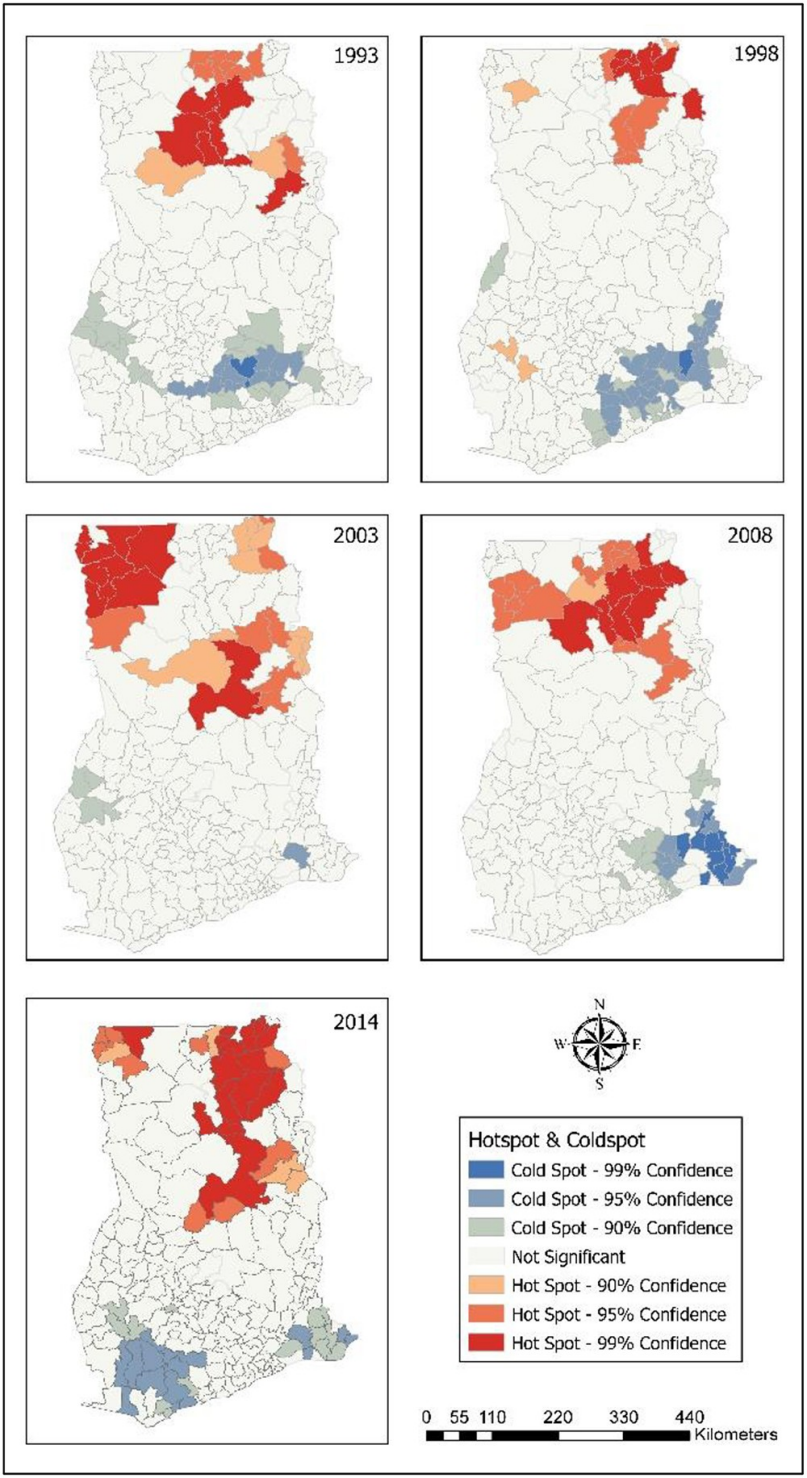
Hot spots of childhood diarrhoea. 1993–Diarrhoea hot spot districts were located in Northern. 1998–Hot spots were found in Upper East, and Northern. 2003–Hot spots in Upper West, and Northern. 2008–Districts identified as hot spots were in Upper East, and Northern. 2014–Districts in Upper West, Upper East, and Northern were hot spots for diarrhoea.

The results from the Local Moran I shows the outliers and clusters. Hotspot/Coldspot analysis furhter Local Morans I by indicating whether the observed spatial clustering of high or low values is more pronounced than one would expect in a random distribution. Statistical results for hotspots/coldspots explains that the hot spots of childhood morbidity were assessed with confidence intervals of: 99%; 95%; and 90% at district levels. The confidence level explains the level of spatial clustering of higher or lower values. In Fig 6, it was found that 16 districts (99% CL), seven districts (95% CL) and four districts (90% CL) were significant hot spots of diarrhoea in 2014. In 2008, eight districts (99% CL), nine districts (95%) and one district (90% CL) were hot spots of diarrhoea. In 2003, 11 districts (99% CL), six districts (95% CL) and 10 districts (90% CL) were hot spots for diarrhoea. Ten districts (99% CL), five districts (95% CL) and three districts (90% CL) were significant hot spots in 1998. For the year 1993, six districts (99% CL), eight districts (95% CL) and two districts (95% CL) were the hot spots. In all the survey years, most diarrhoea hot spot districts were in Northern, Upper East and Upper West. Only hot spot districts with 99% CL are presented in Table 4.

**Table 4:** Hot spot districts of diarrhoea (2014–1993).

Year	Hot spot districts
2014	East Gonja (N)* Sagnarigu (N)* Tamale Metro (N)* Tolon (N)* Savelugu Nanton(N)* Karaga (N)* Gushiegu (N)* Bongo (UE)* Bolgatanga Municipal(UE)* Nabdam (UE)* Talensi (UE)* Bawku West (UE)* Binduri (UE)* Garu-Tempane (UE)* Bawku Municipal (UE)* Sissala West (UW)*
2008	North Gonja (N)* Kumbungu (N)* Savelugu Nanton (N)* Karaga (N)* West Mamprusi (N)* East Mamprusi (N)* Bunkpurugu Yunyoo (N)* Bawku West (UE)*
2003	Nandom (UW)* Lawra (UW)* Jirapa (UW)* Sissala West (UW)* Daffiama Bussie Issa(UW)* Nadowli (UW)* Sissala East (UW)* Wa Municipal (UW)* Wa West (UW)* Wa East (UW)* East Gonja (N)*
1988	East Mamprusi (N)* Chereponi (N)* Bongo (UE)* Bolgatanga Municipal(UE)* Nabdam (UE)* Talensi (UE)* Bawku West (UE)* Binduri (UE)* Garu-Tempane (UE)* Chereponi (UE)*
1993	Mamprugu Moaduri (N)* West Mamprusi (N)* North Gonja (N)* Kumbungu (N)* Tolon (N)* Tamale Metro (N)*

*—99% CL; N—Northern; UE—Upper East; UW—Upper West

**Fig 7:**
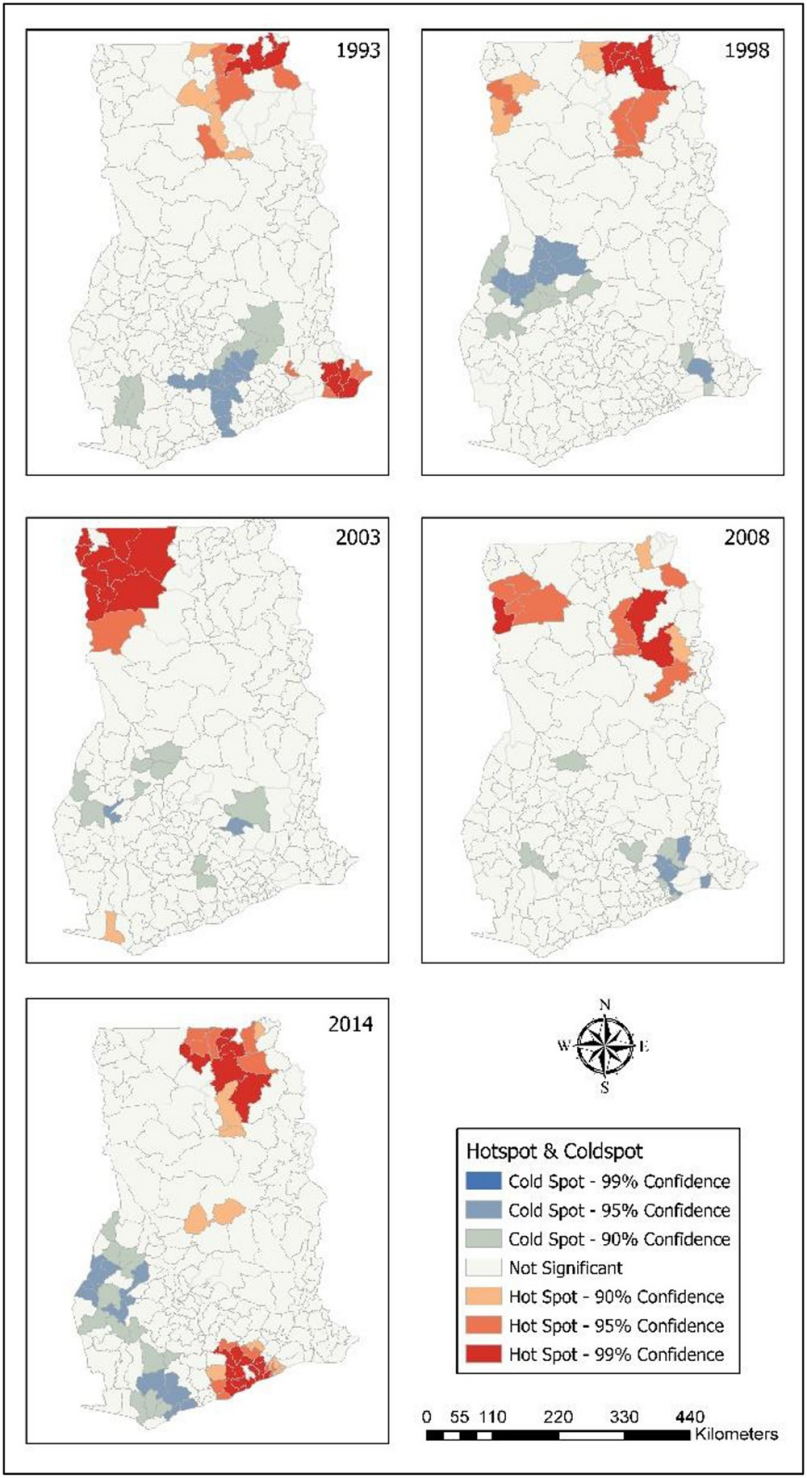
Hot spots of childhood ARI. 1993–Hot spots districts for ARI were in Upper East, and Volta. 1998–Hot spot districts in Upper East, and Northern. 2003–Districts in Upper West were mainly hot spots for ARI. 2008–Districts in Upper West, and Northern were hot spots for ARI. 2014–Hot spot districts were found in Upper East, Northern, Eastern, Accra, and Central.

Fig 7 showed that 18 districts (99% CL), five districts (95% CL), and seven districts (90% CL) were found to be significant ARI hot spots in 2014. Most of the districts affected with ARI were in Upper East, Central and Eastern regions. In 2008, three districts (99% CL), nine districts (90% CL) and two districts (90% CL) were hot spots for ARI. Largely, in 2003, most of the ARI hot spot districts (9) were located in Upper West. In the years 1998 and 1993, Upper East recorded a larger number of ARI hot spot districts (8) compared to the other regions. ARI hot spot districts (99% CL) are summarised in Table 5.

**Table 5:** Hot spot districts of ARI (2014–1993).

Year	Hot spot district
2014	West Mamprusi (N)* Karaga (N)* Bongo (UE)* Bolgatanga Municipal(UE)* Talensi (UE)* Builsa South (UE)* Gomoa West (E)* Agona West Municipal (E)* Birim Central Municipal (E)* West Akim Municipal (E)* Upper West Akim (E)* Nsawam Municipal (E)* Ga West Municipal (GA)* La Nkwantanang/Madina (GA)* Adentan Municipal (GA)* Ledzokuku Krowor Municipal (GA)* Accra Metro (GA)* Ga South Municipal (GA)*
2008	Wa West (UW)* Karaga (N)* Mion (N)*
2003	Nandom (UW)* Lawra (UW)* Daffiama Bussie Issa (UW)* Wa West (UW)* Wa Municipal (UW)* Wa East (UW)* Nadowli (UW)* Sissala West (UW)* Sissala East (UW)*
1998	East Mamprusi (N)* Kassena Nankani East (UE)* Bongo (UE)* Bolgatanga Municipal (UE)* Talensi (UE)* Nabdam (UE)* Bawku West (UE)*
1993	Bongo (UE)* Talensi (UE)* Bawku West (UE)* Garu-Tempane (UE)* Bawku Municipal (UE)*

*-99% CI; N-Northern; UE-Upper East; UW-Upper West; GA-Greater Accra; E-Eastern

**Fig 8:**
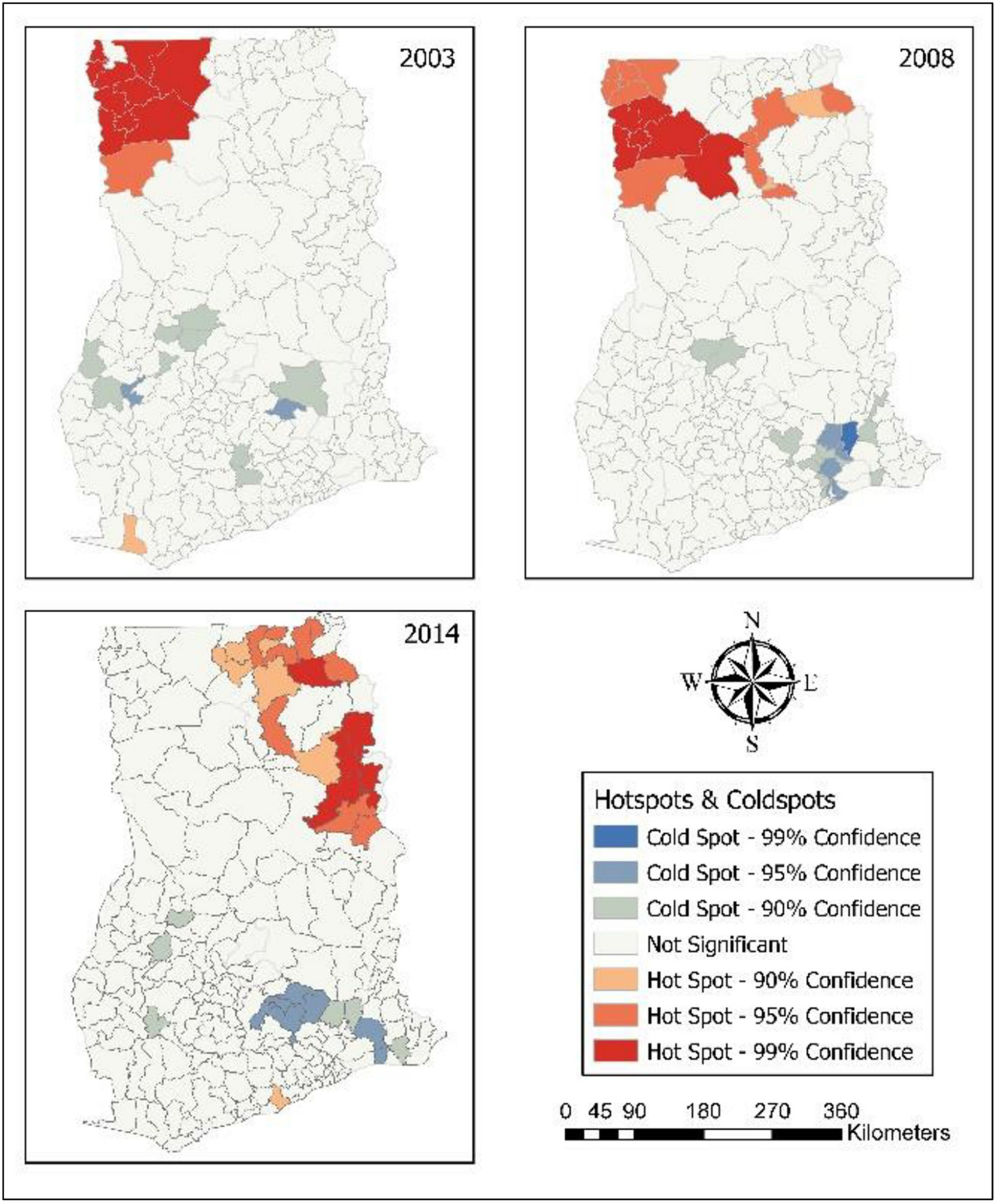
Hot spots of childhood anaemia. 2003–Upper West had most hot spot districts for anaemia. 2008–Hot spot districts were found in Upper West, and Northern. 2014–Hot spot districts were found in Northern.

In relation to anaemia, for the year 2014, all hot spot districts were found in Northern. In 2008, six districts (99% CL), ten districts (95% CL) and two districts (90% CL) were anaemia hot spot districts. Most of them were located in Upper West. All of the anaemia hot spot districts (9) in 2003 were found in Upper West (Fig 8). Table 6 contains anaemia hot spot districts.

**Table 6:** Hot spot districts of anaemia (2014–2003).

Year	Hot spot districts
2014	Saboba (N)* Yendi Municipal (N)* Zabzugu (N)* Nanumba North (N)*
2008	Daffiama Bussie Issa (UW)* Nadowli (UW)* Wa Municipal (UW)* Wa West (UW)* Wa East UW)* West Gonja (N)*
2003	Nandom (UW)* Lawra (UW)* Daffiama Bussie Issa (UW)* Wa West (UW)* Wa Municipal (UW)* Wa East (UW)* Nadowli (UW)* Sissala East (UW)* Sissala West (UW)*

*-99% CI; N-Northern; UE-Upper East; UW-Upper West

**Fig 9:**
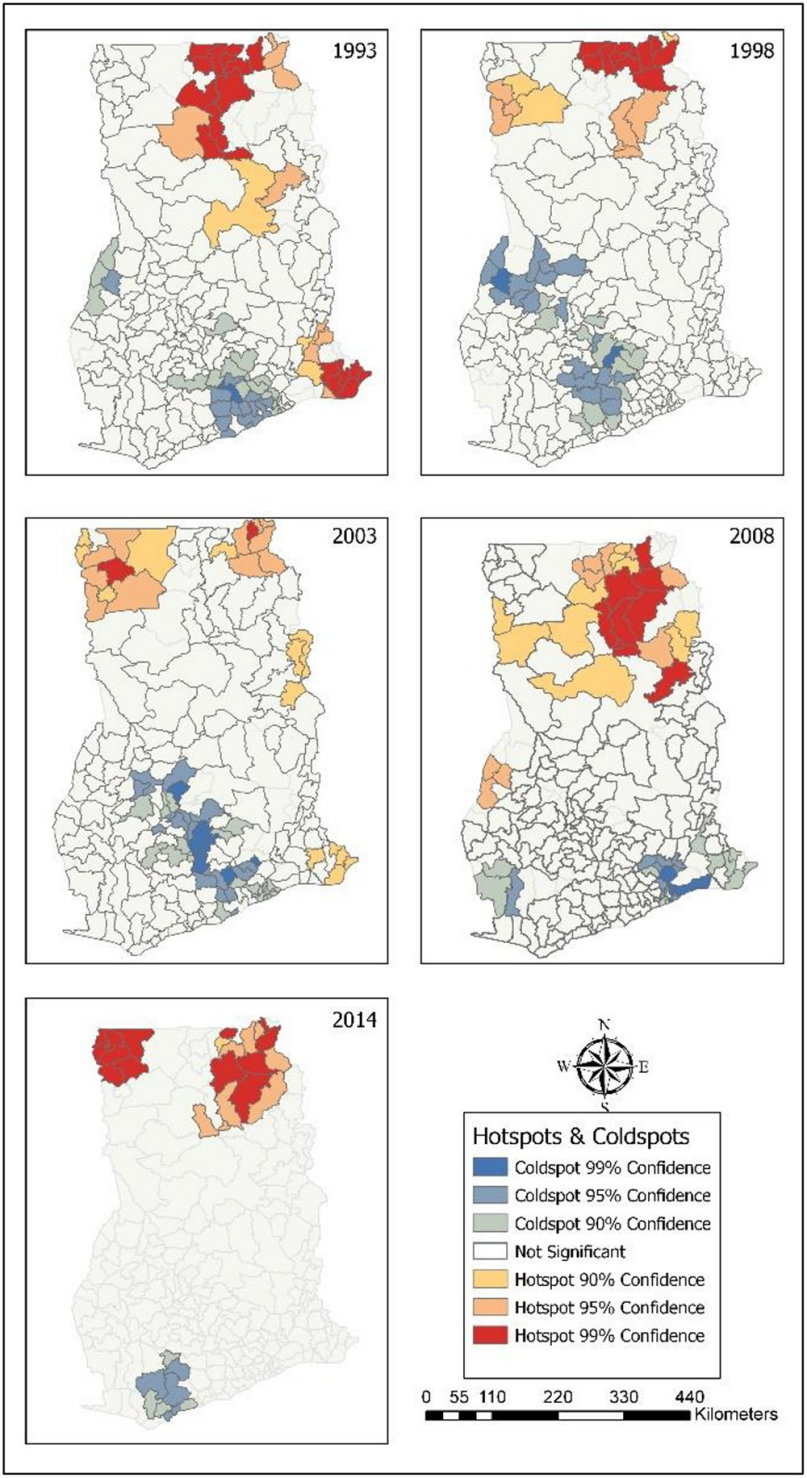
Hot spots of childhood fever. 1993–Hot spot districts for fever were in Upper East, Northern, and Volta. 1998–Upper East, and Northen had hot spot districts. 2003–Hot spot districts were in Upper West, and Upper East. 2014–Hot spots for fever were in Upper West, Upper East, and Northern.

Northern, Upper East and Upper West hosted all of the fever hot spot districts. Fig 9 revealed that seven districts each in Upper East and Upper West, and six districts in Northern that significantly were linked with high occurence of fever in 2014. Nine districts (99% CL), 11 districts (95% CL) and 11 districts (95% CL) were found to be significant hot spot districts in 2008; and Upper West had more fever hot spot districts. In 2003, most of the hot spot districts were found in Upper West (9) and Upper East (6). In 1998, 10 districts (99% CL), five districts (99% CL) and three districts (90% CL) were fever hot districts; and most of them were located in Upper East. For the year 1993, fever hot spot districts were identified: nine districts each in Upper East and Northern; eight districts in Volta; and two districts in Eastern. Fever hot spot districts (99% CL) are shown in Table 7.

**Table 7:** Hot spot districts of fever (2014–1993).

Year	Hot spot
2014	Nandom (UW)* Lawra (UW)* Daffiama Bussie Issa (UW)* Jirapa (UW)* Lambussie Karni (UW)* Sissala West (UW)* Nadowli (UW)* Bongo UE)* Bawku Municipal (UE)* Garu-Tempane (UE)* West Mamprusi (N)* East Mamprusi (N)* Karaga (N)*
2008	Bawku West (UE)* East Mamprusi (N)* West Mamprusi (N)* Kumbungu (N)* Savelugu Nanton (N)* Karaga (N)* Sagnarigu (N)* Tamale Metro (N)* Nanumba North (N)*
2003	Nadowli (UW)* Binduri (UE)*
1988	Kassena Nankana West Builsa North (UE)* Kassena Nankani East (UE)* Bongo (UE)* Bolgatanga Municipal (UE)* Talensi (UE)* Nabdam (UE)* Bawku West (UE)* Binduri (UE)* East Mamprusi (N)*
1993	Kassena Nankani West (UE)* Builsa North (UE)* Kassena Nankani East (UE)* Bongo (UE)* Bolgatanga Municipal (UE)* Talensi (UE)* Bawku West (UE)* Mamprugu Moaduri (N)* West Mamprusi (N)* Kumbungu (N)* Savelugu Nanton (N)* Sagnarigu (N)* Tamale Metro (N)* Keta Municipal (V)* Ketu North (V)* Akatsi South (V)* South Tongu (V)* Central Tongu (V)*

*-99% CI; N-Northern; UE-Upper East; UW-Upper West; V-Volta

## Discussion

The study was designed to mainly reveal hot spots of childhood morbidity in Ghana. In addition, the effect of rural versus urban residence on the occurrence of childhood morbidity was examined. This was done by considering four top morbidities (diarrhoea, ARI, anaemia, and fever) and using five different data sets (1993, 1998, 2003, 2008 and 2014) of GDHS. Children in rural versus urban areas were much significantly associated with reported cases of childhood morbidity. Districts identified to be burdened with each of the childhood morbidities would inform and guide major stakeholders such as Ghana Health Service, District Health Directorates, and health related NGOs to allocated their resources rationally for much desired outcomes.

Most of the hot spot districts for the prevalence of diarrhoea were concentrated in Upper West, Upper East, and Northern. For instance, in the Multiple Indicator Cluster Survey in 2011 [24], Northern Region recorded the highest prevalence of childhood diarrhoea. Records of high prevalence of diarrhoea in this part of the country may be attributed to environmental factors such as insufficient access to potable water and poor sanitation. These factors expose children to diseases; and diarrhoea is the most common. A study conducted by Cheng et al. [25] documented that 50 percent of the population in Northern Region has access to improved drinking water. And in Savelugu-Nanton, less than 50 percent of its inhabitants had access to improved drinking water. Again open defecation is highly practiced among districts in the region despite the implementation of interventions such as Community-Led Total Sanitation [26]. Open defecation encourages the transfer of pathogens from faeces to food and water systems through run-off and houseflies which has the potential to cause diarrhoea in the in the region hence, Savelugu-Nanton. Osei (2017) attests to this poor sanitation as the major causes of diarrhoea in the region. These conditions cited in Northern are similar to those in Upper East and Upper West. Therefore, it is likely that these other districts are faced with sanitation and water challenges.

Additionally, hot spots for ARI were commonly clustered in Upper East, Upper West, and Northern. Some districts in Accra, in 2014, were equally identified as hot spots for episodes of ARI. The top rated hot spot for childhood ARI was Accra Metro (the capital city) in the Greater Accra Region. In densely populated urban areas, like Accra, overcrowding, vehicular fumes and industrial waste (gasses) could cause ARI among children. Children in urban areas might be more exposed to pollutants such as carbon monoxide, nitrogen oxide and sulfur dioxide emitted by cars and industries. Another reason for high rates of ARI in Accra could be attributed to low level of vegetation and tree life which can serve as air filters as the area is more of a ‘concrete jungle’. For the occurrence of ARI in the other districts which are more in rural settings may be due to cooking arrangements where children are exposed to harmful gasses from fuelwood.

Further, hot spot districts identified for prevalence of anaemia were located along the north-eastern corridor, and north western portions of the country. And the unavailability the required foods for children to prevent anaemia may not be readily available in the hot spot districts. In a study by Ewusie et al. [5], they revealed that Upper East Region had the highest prevalence rate of anaemia. During the surveys, blood samples were taken from children to test for anaemia and a number of children who were anaemic had low haemoglobin levels less than 11 grams per deciliter (g/dl) [12]. This, accordingly, is caused by the kinds of low-quality foods children consume that predispose them to reduced red blood cells and consequently decreased levels of haemoglobin [27]. To avert this, it is recommended that children should consume additional to basic cereals such as maize, millet, sorghum, and soy [28]. This, therefore, suggests that most children in the identified hot spot districts consumed poor diets. Thus, diets that do not contain essential quantities of iron and micronutrients required to boost haemoglobin levels. Aside the aforementioned reasons, there are food insecurity issues in the districts and this might have compounded dietary deficiencies among children, thereby resulting in high prevalence of anaemia among children [29].

In the study, there were also districts identified as hot spots for fever; mainly in the north-western and north-eastern parts of the country. Most of the hot spot districts were situated in Upper West and Northern regions across the survey years. High rates of fever in these locations could be as a result of high malaria cases; since fever is a common symptom of malaria among children. Other infections caused by viruses and bacteria among children could have also contributed to high episodes of fever in the identified hot spot districts [30].

The hot spot districts identified are assumed to be homogeneous. Identification of specific target localities such as households, and more socio-economic and environmental factors within districts may further inform the formulation and implementation of interventions. Notwithstanding the stated limitations, this study has importantly examined the spatial patterns and hot spots of four top childhood morbidities in Ghana using districts as the main reference at five different time points. These findings should be interpreted with caution since seasonality of weather conditions were not part of the study variables.

## Conclusion

Diarrhoea and fever hot spots are in the north-western, north-eastern and middle portions of the country. For ARI, hot spots are in the north-eastern, middle, and along the coast. Childhood anaemia cases are common in the north-eastern corner, and hot spots for fever are mainly in the northern divide of the country. To minimize childhood morbidity in Ghana, various Ghana Health Directorates could design context-based interventions to target hot spot districts within their administrative areas. However, further geospatial studies on households within districts would be more beneficial for various local health offices to adequately target populations at risk.
